# Biological Networks for Predicting Chemical Hepatocarcinogenicity Using Gene Expression Data from Treated Mice and Relevance across Human and Rat Species

**DOI:** 10.1371/journal.pone.0063308

**Published:** 2013-05-30

**Authors:** Reuben Thomas, Russell S. Thomas, Scott S. Auerbach, Christopher J. Portier

**Affiliations:** 1 Division of Environmental Health Sciences, School of Public Health, University of California, Berkeley, California, United States of America; 2 The Hamner Institutes for Health Sciences, Research Triangle Park, North Carolina, United States of America; 3 Biomolecular Screening Branch, National Toxicology Program, National Institute of Environmental Health Sciences, Research Triangle Park, North Carolina, United States of America; 4 National Center for Environmental Health and Agency for Toxic Substances and Disease Registry, United States Centers for Disease Control and Prevention, Atlanta, Georgia, United States of America; IIT Research Institute, United States of America

## Abstract

**Background:**

Several groups have employed genomic data from subchronic chemical toxicity studies in rodents (90 days) to derive gene-centric predictors of chronic toxicity and carcinogenicity. Genes are annotated to belong to biological processes or molecular pathways that are mechanistically well understood and are described in public databases.

**Objectives:**

To develop a molecular pathway-based prediction model of long term hepatocarcinogenicity using 90-day gene expression data and to evaluate the performance of this model with respect to both intra-species, dose-dependent and cross-species predictions.

**Methods:**

Genome-wide hepatic mRNA expression was retrospectively measured in B6C3F1 mice following subchronic exposure to twenty-six (26) chemicals (10 were positive, 2 equivocal and 14 negative for liver tumors) previously studied by the US National Toxicology Program. Using these data, a pathway-based predictor model for long-term liver cancer risk was derived using random forests. The prediction model was independently validated on test sets associated with liver cancer risk obtained from mice, rats and humans.

**Results:**

Using 5-fold cross validation, the developed prediction model had reasonable predictive performance with the area under receiver-operator curve (AUC) equal to 0.66. The developed prediction model was then used to extrapolate the results to data associated with rat and human liver cancer. The extrapolated model worked well for both extrapolated species (AUC value of 0.74 for rats and 0.91 for humans). The prediction models implied a balanced interplay between all pathway responses leading to carcinogenicity predictions.

**Conclusions:**

Pathway-based prediction models estimated from sub-chronic data hold promise for predicting long-term carcinogenicity and also for its ability to extrapolate results across multiple species.

## Introduction

There is push to use a broad array of biological data in toxicity testing to improve reliability and provide reasonably quick indications of animal and human toxicity of chemical compounds. The National Toxicology Program (NTP) 's High Throughput Screening (HTS) [1] and the Environmental Protection Agency's ToxCast programs [2] are efforts in this direction. These efforts have been initiated in light of the fact that for a lot of the 100,000 chemicals in commerce in the U.S. and Europe, information on toxicity is extremely limited [3]. Data provided from the long term chronic testing of chemicals on animals, like those from NTP's two-year cancer bioassay though invaluable in providing carcinogenicity information *in vivo* over a wide range of animal tissues and across multiple doses, is not suitable when one needs to rapidly identify potential harm from chemical exposure. Over the past approximately 40 years, only around 600 chemicals have been tested by the NTPs in their two-year cancer bioassay and only about 5–6 times this number have been tested worldwide.

The goal of toxicity testing is ultimately to protect human health. Even though the two-year cancer bioassay is performed on rats and mice, there is considerable data supporting its use to screen for carcinogens in humans [4]. The HTS and ToxCast programs are performed on cell lines; these can be both human and animal and thus provide a logical path for extrapolation between species. However, cell lines are distantly removed from a functional whole organism so it becomes necessary to extrapolate the *in vitro* results from the various cell lines to the human *in vivo* context.

An alternative to the cell-based assays in the HTS and ToxCast programs is the use of short term molecular data (typically gene expression) from exposed animals or humans to predict future toxicity or carcinogenicity. Others have used gene expression data based classifiers that would distinguish a toxin or carcinogen from a non-toxin or non-carcinogen [5], [6], [7], [8], [9], [10], [11], [12], [13], [14]. These representative examples demonstrate different approaches to predictive toxicology using gene expression data obtained from rodents dosed over a period of time ranging from a day to 90 days. In all of the published studies, chemicals were classified in a binary fashion (for example, attempts were made to classify chemicals as being carcinogenic or not). Such an approach underutilizes the results from the chronic studies in light of the fact that a continuous statistic was used to evaluate the data originally (e.g. p-value from the Fisher exact test or the trend statistic from the Poly-3 test [15]) and a considerable amount of other biological information was brought to bear on a final evaluation. This leads to obvious loss of information regarding the differential toxicity of different compounds at their relevant doses.

A characteristic of gene expression data is the very large features (genes) to samples (animals) ratio. This characteristic contributes to the lack of identification of robust classifiers as demonstrated by [16] – multiple classifiers give the same prediction accuracies which quite often are 100 percent. This could mean that the data was over-fit or there are correlations between genes that allow for alternate sets of genes to server as predictors. In the second scenario of correlated gene expressions, this would suggest the use more of mechanistically-relevant prediction models where the data is examined at the biological pathway level rather than at the gene level.

In order to gauge quantitative predictive accuracy of a pathway-based prediction model we obtained gene expression data from the livers of mice dosed with 26 chemicals over a period of 90 days. All the 26 chemicals were tested in a two-year bioassay in the same strain of mice dosed via the same respective routes. Thus, the differential liver carcinoma and adenoma rates between treated and control animals from the results of the two-year bioassays are known. Using the data, we derived pathway-based models to predict the differential tumor rates at the end of a two-year bioassay. The use of a pathway-based model would result in a reduction of a model with around 6000 gene features to one with around 200 pathways in the KEGG database [17], [18], [19]. The model derived using the mouse data was then used to predict carcinogenicity in appropriately chosen scenarios in rats and humans.

## Materials and Methods

### Mice chemicals, animals and treatments

The chemical treatments used in the experiments on female B6C3F_1_ mice are summarized in Table 1. Note the treatments are the same as the maximum tolerable dose (MTD) used for the corresponding chemical in the two-year bioassay. Treatments involving lower doses of a subset of 5 chemicals from Table 1 are provided in Table 2. More detailed descriptions of the chemicals, treatments and gene expression analysis are provided in the Text S1.

**Table 1:** Treatment groups and abbreviations used in the 90 day exposure to the 26 chemicals and corresponding vehicle controls used in this study.

Chemical	Short Name	NTP No.	Route^a^	Dose	Ames Assay	Liver Tumors^b^
1-Amino-2,4-dibromoanthraquinone	ADBQ	383	F	20,000 ppm	+	Yes
Benzofuran	BFUR	370	GC	240 mg/kg	−	Yes
Methylene Chloride	MECL	306	I	4,000 ppm	+,−,+	Yes
N-Methylolacrylamide	MACR	352	GW	50 mg/kg	−	Yes
1,5-Naphthalenediamine	NAPD	143	F	2,000 ppm	+	Yes
Tris(2,3-dibromopropyl)phosphate	TDPP	76	F	1,000 ppm	−	Yes
2,2-Bis(bromomethyl)-1,3-propanediol	BBMP	452	F	1,250 ppm	+,+,−	No
1,2-Dibromoethane	DBET	86	GC	62 mg/kg	+	No
Ethylene Oxide	ETOX	326	I	100 ppm	+	No
Naphthalene	NPTH	410	I	30 ppm	−	No
Vanadium Pentoxide	VANP	507	I	2.0 mg/m^3^	−	No
Benzene	BENZ	289	GC	100 mg/kg	−	Eq^c^
Coumarin	COUM	422	GC	200 mg/kg	+	Eq^c^
1,2,3-Trichloropropane	TCPN	384	GC	60 mg/kg	+,+	Yes
1,4-Dichlorobenzene	DCBZ	319	GC	600 mg/kg	−	Yes
Propylene glycol mono-*t*-butyl ether	PGBE	515	I	1,200 ppm	+	Yes
Tetrafluoroethylene	TFEL	450	I	1,250 ppm	NA	Yes
2-Chloromethylpyridine hydrochloride	CMPH	178	GW	250 mg/kg	+	No
Diazinon	DIAZ	137	F	200 ppm	−	No
Iodoform	IODO	110	GC	93 mg/kg	+,+	No
Malathion	MALA	24	F	16,000 ppm^d^ (14,800 ppm)	−	No
N-(1-naphthyl) ethylenediamine dihydrochloride	NEDD	168	F	3,000 ppm (2,000 ppm)^e^	+	No
4-Nitroanthranilic acid	NAAC	109	F	10,000 ppm	+,+	No
Pentachloronitrobenzene	PCNB	61	F	8,187 ppm	−	No
Tetrafluoroethane	TFEA	---f	I	50,000 ppm	NA	No
Trichlorofluoromethane	TCFM	106	GC	3,925 mg/kg	−.−	No
Air	ACON		I			
Corn oil	CCON		GC			
Feed	FCON		F			
Water	WCON		GW			

a I  =  inhalation; F  =  feed; GC  =  gavage, corn oil (5 ml/kg); GW  =  gavage, deionized water (5 ml/kg).

b The results for liver tumors were based on a *p = 0.01* threshold for combined increase in adenomas or carcinomas.

c The results for liver tumors in this study were considered equivocal or borderline significant. Combined increase in hepatocellular adenomas or carcinomas resulted in *p = 0.075* and *p = 0.084* for benzene and coumarin respectively.

d Due to signs of toxicity, the 16,000 ppm dose was reduced to 0 ppm on day 9 for a period of 2 days. The dose was raised to 8,000 ppm for a period of 9 days and returned to 16,000 ppm for the remainder of the study. The time weighted average dose was 14,800 ppm.

e The initial dose of 3,000 ppm was reduced to 2,000 ppm in week 2 of the study due to taste aversion and weight loss. The 2,000 ppm dose is the same as the low dose in the original bioassay.

f Chemical not evaluated by the NTP. Bioassay performed by Alexander *et* *al.* [20].

**Table 2:** Dose response treatment groups and abbreviations used in the 90 day exposure with the results from the NTP rodent cancer bioassay.

Chemical	Short Name	NTP No.	Route^a^	Dose	Dose Tested in NTP Bioassay	Liver Tumors
Methylene Chloride	MECL5	306	I	4,000 ppm	Yes	Yes
	MECL4			3,000 ppm	No	
	MECL3			2,000 ppm	Yes	Yes
	MECL2			500 ppm	No	
	MECL1			100 ppm	No	
Naphthalene	NPTH5	410	I	30 ppm	Yes	No
	NPTH4			20 ppm	No	
	NPTH3			10 ppm	Yes	No
	NPTH2			3 ppm	No	
	NPTH1			0.5 ppm	No	
1,2,3-Trichloropropane	TCPN5	384	GC	60 mg/kg	Yes	Yes
	TCPN4			40 mg/kg	No	
	TCPN3			20 mg/kg	Yes	No
	TCPN2			6 mg/kg	Yes	No
	TCPN1			2 mg/kg	No	
Propylene glycol mono-*t*-butyl ether	PGBE5	515	I	1200 ppm	Yes	Yes
	PGBE4			800 ppm	No	
	PGBE3			300 ppm	Yes	No
	PGBE2			75 ppm	Yes	No
	PGBE1			25 ppm	No	
1,4-Dichlorobenzene	DCBZ5	319	GC	600 mg/kg	Yes	Yes
	DCBZ4			500 mg/kg	No	
	DCBZ3			400 mg/kg	No	
	DCBZ2			300 mg/kg	Yes	No
	DCBZ1			100 mg/kg	No	

The vehicle controls were the same as given in Table 1.

a I  =  inhalation; GC  =  gavage, corn oil (5 ml/kg).

### Rat liver gene expression data

To address the question of species extrapolation, gene expression and tumor data from chemical exposures in Fischer 344 rats were obtained from the literature [5]. The chemical treatments are summarized in Table S1. Additional information is provided in the Text S1. Normalized probe intensity data for each treatment were grouped separately with the control and normalized using the quantile normalization function in MATLAB (2008a, The MathWorks, Natick, MA).

### Human gene data

The pathology review of the treated mice after 90 days of exposure to hepatocarcinogens did not identify liver tumors (see Text S1). Hence, the 90 day gene expression patterns are potentially reflective of a pre-neoplastic state in the case of the chemicals that are known hepatocarcinogens (as characterized by the 2 year bioassay). We considered known risk factors for various human diseases including liver cancer. Both genetic (i.e., single nucleotide polymorphisms) and non-genetic (i.e., disease) risk factors were considered.

Genetic risk factors in terms of single nucleotide polymorphisms associated with various human diseases including liver cancer are tabulated on databases like the Genetic Association Database [20]. Genes having polymorphisms associated with various human cancers,Alzheimer's disease and Schizophrenia were downloaded from the database. Identifying non-genetic risk factors for liver cancer has been an active research topic [21], [22], [23], [24], [25], [26], [27], [28]. Among the main factors contributing to the risk are Hepatitis C and B virus [22], [24], [25], cirrhosis [21] and diabetes [27], [28]. The risks of liver cancer associated with cirrhosis induced by Hepatitus C virus was estimated to be comparable with cirrhosis induced by non-alcoholic steatohepatitis (NASH) [29]. In fact NASH is correlated with characteristics of metabolic syndrome like obesity and diabetes. Various human gene expression datasets associated with these risk factors are identified on the Gene Expression Omnibus (GEO) database [30]. The processed and normalized gene expression data were used as is from the database except for cases where the data were presented as intensity values. In these cases, the intensity values were log_2_-transformed. The entire set of data sets associated with risk factors for liver cancer is summarized in Table 3.

**Table 3:** Human data sets associated with risk for liver cancer that were used for carcinogenicity predictions.

ID^a^	Risk factor	Treatment comparison	Experiment description
GDS2239	HCV	3 HCV core protein induced vs 3 control	Hepatitis C virus core protein effect on hepatocyte cell line
GDS3347	Type 2 diabetes	10 normal and 10 diabetic	Type 2 diabetes: cultured myotubes
GDS3656	Type I diabetes	11 normal and 11 type I diabetic	Folic acid effect on endothelial progenitor cells of type 1 diabetes patients
GSE10356_AC	Alcoholic cirrhosis	8 control and 7 alcoholic cirrhosis	Post-alcoholism and post-hepatitis C cirrhosis
GSE10356_HC	HCV cirrhosis	8 control and 7 HCV cirrhosis	Post-alcoholism and post-hepatitis C cirrhosis
GSE15331	HCV	6 HCV −ve and 24 HCV +ve	mRNA expression in human hepatitis c virus (HCV) liver biopsy samples
GSE15653	Type 2 diabetes	5 control and 5 obese and well-controlled DM	Expression data from liver of obese (with or without type 2 diabetes) and lean human subjects
GSE16415	Type 2 diabetes	5 normal and 5 diabetic	Genome wide gene expression profiling of visceral adipose tissue among Asian Indian diabetics
GSE20948_12 hrs	HCV	3 Huh7 cells_JFH-1 Infected after 12 hours vs 3 Huh7 cells_Mock Infected after 12 hours	Effect of Hepatitis C Virus Infection on Host Gene Expression
GSE20948_18 hrs	HCV	3 Huh7 cells_JFH-1 Infected after 18 hours vs 3 Huh7 cells_Mock Infected after 18 hours	Effect of Hepatitis C Virus Infection on Host Gene Expression
GSE20948_24 hrs	HCV	3 Huh7 cells_JFH-1 Infected after 24 hours vs 3 Huh7 cells_Mock Infected after 24 hours	Effect of Hepatitis C Virus Infection on Host Gene Expression
GSE20948_48 hrs	HCV	3 Huh7 cells_JFH-1 Infected after 48 hours vs 3 Huh7 cells_Mock Infected after 48 hours	Effect of Hepatitis C Virus Infection on Host Gene Expression
GSE23343	Type 2 diabetes	7 normal and 10 diabetic	Expression data from human liver with or without type 2 diabetes
SNP set	Genes with Single Nucleotide Polymorphisms (SNPs) associated with *human bladder, brain, breast, cancer, cervical, colorectal, endometrial, esophageal, gastric, head and neck, liver, lung, lymphoma, lymphoma-Hodgkins disease, ovarian, pancreatic, prostate, renal, skin-non melanoma, testicular, thyroid, leukemia, leukemia-childhood acute lymphoblastic* cancers and *alzheimer's* and *schizophrenia* diseases		

a The first 13 data sets with IDs beginning GDS- or GSE- represent gene expression data obtained from GEO database (http://www.ncbi.nlm.nih.gov/geo/, accessed June 2009). The gene polymorphism data associated with various human diseases are obtained from the GAD database (http://geneticassociationdb.nih.gov/, accessed June 2009).

### Biochemical pathways used in the analysis

The biochemical pathways used in the analysis in this paper were obtained from the Kyoto Encyclopedia of Genes and Genomes (KEGG) Pathway database [17], [18], [19]. The data for the set of genes involved in each of the pathways and their associated interactions were downloaded via the KEGG API. All pathways in the database were used. The list of 216 pathways that were homologous across human, mice and rats species, along with their mouse KEGG ids are reported in Table S2.

### Structurally enhanced pathway enrichment analysis (SEPEA)

SEPEA, a network based pathway enrichment method described in detail in [31], was used to evaluate the linkage between the gene expression data and the KEGG pathways. Unlike traditional pathway enrichment methods that treat pathways as sets of genes, SEPEA treats pathways as networks of interacting proteins and/or enzymes. The genes corresponding to the proteins in the signaling network are given more weight according to whether they are at the receptor or the terminating end of the pathway that typically signals for transcription in a number of genes. Further, pathways where the perturbed genes are close relative to each other on the associated network are modeled as being more likely to be affected than pathways where the perturbed genes occur further apart over the network. There were three analytic methods described in [31]; in the work described here, SEPEA_NT2 is used [31] for the gene expression analysis and SEPEA_NT3 for the gene polymorphism data. The goal of the enrichment analysis is to assign significance (in terms of p-values) to all chosen KEGG [17], [18], [19] human, rat and mouse pathways for the increased likelihood of being affected in the livers of the treated animals over the matched controls. The significance obtained by SEPEA_NT2 were based on 5×10^4^ randomizations (see [31] for details). For the analysis performed here, the SEPEA p-value was converted to a z-score, *Z_ij_* using the equation,
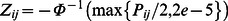
(1)where Φ refers to the standard normal distribution and *P_ij_* denotes the p-value obtained using SEPEA for pathway *j* as a result of the mice being dosed with chemical *i* at a given dose or human data set *i*.

### Evaluation of carcinogenicity for mice chemical data

The carcinogenicity rate of a chemical is defined using the survival-adjusted proportions of animals treated with the chemical that developed liver adenomas or carcinomas and the corresponding proportion for the control animals at the end of the two-year bioassay. The data for all chemicals except tetrachloroethane [32] were obtained from their respective technical reports for the two-year cancer bioassay developed by the NTP(see Text S1 for reference to the NTP technical reports). The poly-3 survival-adjusted numbers [15], [33] were used when available, else the survival adjustment provided in the technical reports were used. The poly-3 statistic, *z_i_*, for chemical *i* used in this analysis is defined as,
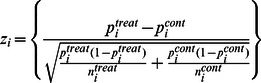
(2)where *n_i_^treat^* and *p_i_^treat^* are the survival adjusted number and proportion, respectively of animals treated with chemical *i* that developed adenomas or carcinomas, *n_i_^cont^* and *p_i_^cont^* are the corresponding terms for the control animals. To avoid outlier effects in the analysis, *z_i_* = *z_2e−5_* was used when *z_i_*≥*z_2e-5_* where *z_2e−5_* is the critical value of the standard normal distribution corresponding to 2×10^−5^ significance.

### Prediction model relating carcinogenicity to either pathway features

The critical hypothesis of this manuscript is that the observed changes in gene expression manifest at the pathway level have adequate information to predict future carcinogenicity. However, it is not obvious what mathematical functional form relates the observed changes in pathways to the carcinogenicity predictions.

Specifically, the poly-3 statistic for a chemical obtained from the two-year cancer bioassays is assumed to be functionally related to the 90 day gene expression-based perturbation of the pathways (obtained from the SEPEA analysis):
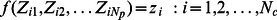
(3)where *N_p_* represents the number of pathways (216, in the analysis in this paper), *N_c_* the number of chemicals (26 chemicals were tested in mice). *z_i_* is given by Equation (2) and *Z_ij_* is given by Equation (1) for the pathway-specific predictor. Note, the model is fit to data sets corresponding to chemical treatments in Table 1.

The framework of the so-called Super Learner [34] coded in the SuperLearner package [35] in R [36] provides a reasonable way of evaluating alternate functional forms in a cross-validation framework. The functional forms tried include tree-based methods (random forests [37], bagging, conditional tree forests), support vector machines, loess polynomial regression, bayes generalized linear models, sparse partial least squares regression and neural-networks – the book [38] describes details of all of the these algorithms except random forests. Unless otherwise specified the default settings of these learning algorithms in the SuperLearner package are used. The SuperLearner algorithm that is based on the optimal continuous combination of the predictions of the other algorithms and the Discrete SuperLearner that picks the best predictor at each fold of cross-validation were used. The performances of these algorithms were evaluated in terms of their cross-validated risk [34].

Five-fold cross-validation was chosen where the chemicals in each of the five folds were fixed (Table S3) and chosen so that distribution of high and low carcinogenicity values in each fold was more or less the same. Predictions for all the data sets were based on this 5-fold cross validation framework. In order to get ‘honest’ predictions for the chemicals with data from mice, because these chemical data are used to make predictions for the very same set of chemicals, two levels of 5-folds cross-validation was implemented using the *CV.SuperLearner* function implemented in the SuperLearner package. Specifically, the predictions for the chemicals in each of the 5 test sets (corresponding to the 5-folds) are derived from an additional 5-fold cross-validation of the remaining 20 or 21 chemicals in the 5 training tests.

### Evaluation of the results of the predictions

The two year cancer bioassay carcinogenicity calls of the chemicals used in the mice and rats experiments along with the continuous predictions from the model (Equation (3)) derived using the mouse data are used to generate receiver-operator (ROC) curves using the pROC package [39] in R [36]. The carcinogenicity calls (carcinogenic or non-carcinogenic) are based on a significance p-value threshold of 0.01 using the statistic given in Equation (2)) Myristicin and isosafrole were untested chemicals among the rat data and predictions for these were not used in generating the ROC curves. For the case of the human data, all gene expression data sets associated with risks for liver cancer were considered positive for carcinogenicity. The data set of gene polymorphisms associated with liver cancer was also considered positive while all other gene polymorphism data sets were considered negative. The ROC curve naturally defines notions of false positives and false negatives at chosen levels of specificity and sensitivity on the ROC curves. Additionally the area-under-the-curve (AUC) measures [40] of the ROC curves.

## Results, Discussion and Conclusions

### Choice of prediction algorithm

Different prediction models were evaluated in terms of their 5-fold cross-validated risk for fitting the model given by Equation (3) (see Table S4). Random forest and support vector machines using all the pathway features displayed the lowest cross-validated risk. Random forests [37] (denoted by *SL.randomForest.1_All* in Table S4) were because they provide a rigorous metric called *importance* for each of the biochemical pathways used in the prediction model. The *importance* of a pathway is a measure of how much it contributes to increases the accuracy of the predictions.

### Receiver-operator curves for the predictions in the three species

**Figure 1:**
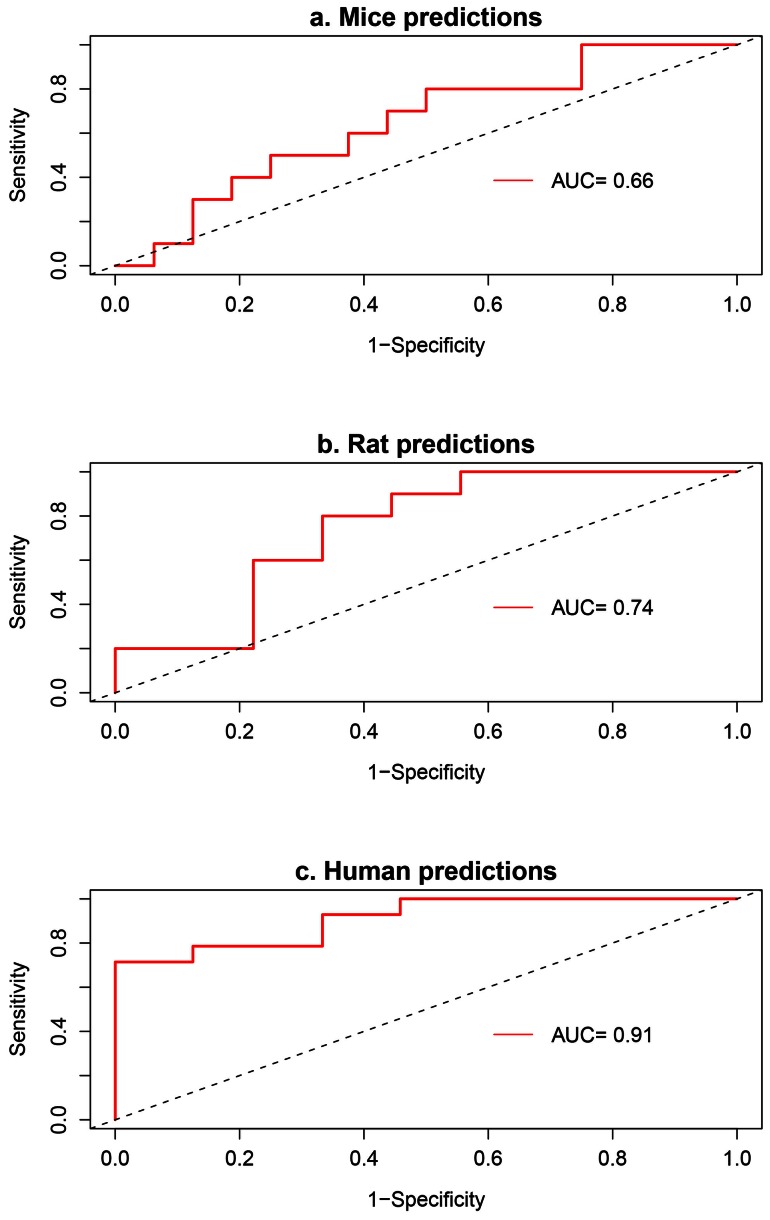
Receiver-operator characteristics (ROC) curves of carcinogenicity predictions using the pathway-based prediction models across three species. (a. Mice, b. Rats and c. Humans) The legend in the sub-plots provides the area-under-the-curve (AUC) for the corresponding ROC curve. The curves for mice, rats and humans are based on datasets corresponding to those in Tables 1, Table S1 and Table 3 respectively.

The model derived using the mouse data was used to predict the carcinogenicity in mice, rats and humans for the corresponding data sets. In this manuscript, the predicted values were treated as proxies for the continuous NTP carcinogenicity values in Equation (2). The receiver-operator curves for the three species are shown in Figure 1. The Area-Under-the-Curve (AUC) metric for both mice and rats (0.66 and 0.74) are reasonably good while it was 0.91 for humans. This is a very encouraging result suggesting the utility of extrapolation of stated mice results to the case of humans. The fact that the rat and human prediction performances were better than that for the mice may seem surprising and counter-intuitive. However, it should not be. For the data sets under consideration, the performance of predictions from test sets (in this case those from rats and humans) using the inferred prediction model depends on how closely the majority of positive (negative) test sets were to a majority of the elements of the positive (negative) training set (in this case those from mice). The ‘closeness’ here is measured in the pathway-response feature space. Therefore, the mechanisms of hepatocarcinogenicity implied by the pathway responses of the 26 chemicals in mice were enough to adequately cover the corresponding mechanisms seen in the data sets from rats and humans.

The false positives and false negatives in each of the three species at a level of specificity between 56–75% (i.e., comparable Type I error rate) is summarized are Table 4. A further discussion of the identified false positives and false negatives is provided in Text S1.

**Table 4:** False positives and false negatives predictions of predictors across the three species at appropriately chosen points on the receiver-operator curves in Figure 1.

ROC curve	False positives	False negatives	Sens	Spec
Mice	naac, iodo, nedd, coum, mala, pcnb, dbet	abdq,macr,tdpp,tfel	0.6	0.56
Rats	APAP, TYP, VtC	ESG_LOW,SAF_LOW,MEG_LOW	0.7	0.67
Humans	cancer of the cervix, endometrium, esophogus ,stomach, head and neck, lung, , ovary, testicle and lymphoma, Hodgkins disease	GSE16415	0.93	0.58

This paper presents results only for pathway-based prediction models. Gene-based prediction models were also evaluated and they showed a similar performance in terms of the AUC metric (data not shown) [12]. Evaluated gene-based models for predicting lung carcinogenicity using the same set of 26 chemicals as used in this paper. The average AUC metric they observed using a range of learning models was around 0.7.

### Features of the prediction models

The importance measures for each of the pathway-based features are reported in Table S2. The top fifteen pathways ranked by their importance measures output from the random forests learning algorithm are provided in Table 5. From the KEGG pathway database [17], [18], [19], the broad categories in which each of the pathways lie are also provided. Among these broad categories, altered fatty acid metabolism is associated with liver steatosis, leading to steatohepatitis and subsequently to an inflamed liver, liver cell death including apoptosis, inflammation, hepatocellular regeneration, stellate cell activation, and fibrogenesis, events that culminate in cirrhosis and liver cancer [41], [42]. This could also explain the reason why the gene expression data based on non-genetic risk factors like obesity, cirrhosis and diabetes were predictive of chemical-induced hepatocarcinogenesis. Calcium signaling is associated proliferating cells [43] and also along with cytochrome c in programmed cell death, apoptosis [44], [45]. Perturbed glycan synthesis has been found in ovarian cancer [46] and human mammary, colon [47], hepatic [48], [49], and glial tumors [50]. Other pathways included those associated with altered gene transcription and translation, xenobiotic, vitamin and carbohydrate metabolism. A clustergram of the z-transformed SEPEA p-values for all the pathways across the 26 chemicals used for the mouse data is shown in figure S1 (see Text S1 for details of generation of the clustergram). The lack of predictability especially for the case of mice (because prediction was based on mice data) suggests plausible new features that are not captured by the the pathway-based ones or that the set of 26 chemicals was not diverse enough in terms of having sufficient number of chemicals with alternate mode-of-actions. Some pathways such as Amyotrophic lateral sclerosis (ALS) may have relatively large importance measures but may have no direct significance to the biological processes in the liver. The interpretation one should have in such cases is that the responses of the set of genes associated with ALS disease process in the liver are relevant to the prediction of hepatocarcinogenicity.

**Table 5:** Top 15 pathways of the fitted prediction model.

KEGG pathway	Broad pathway category	Importance Score
Drug metabolism	Xenobiotics Biodegradation and Metabolism	1.62
Glyoxylate and dicarboxylate metabolism	Carbohydrate metabolism	1.15
Pentose and glucuronate interconversions		1.13
Ascorbate and aldarate metabolism		1.13
O-Mannosyl glycan biosynthesis	Glycan Biosynthesis and Metabolism	1.02
Apoptosis	Cell Growth and Death	1.01
Calcium signaling pathway	Signal Transduction	0.99
Retinol metabolism	Metabolism of Cofactors and Vitamins	1.45
Thiamine metabolism		1.00
Ribosome	Transcription and Translation	1.11
RNA polymerase		1.01
Arachidonic acid metabolism	Lipid metabolism	1.93
Steroid hormone biosynthesis		1.09
Glycosphingolipid biosynthesis - globo series		1.04
Amyotrophic lateral sclerosis (ALS)	Neurodegenerative Diseases	1.29

### Dose-response predictions

**Figure 2:**
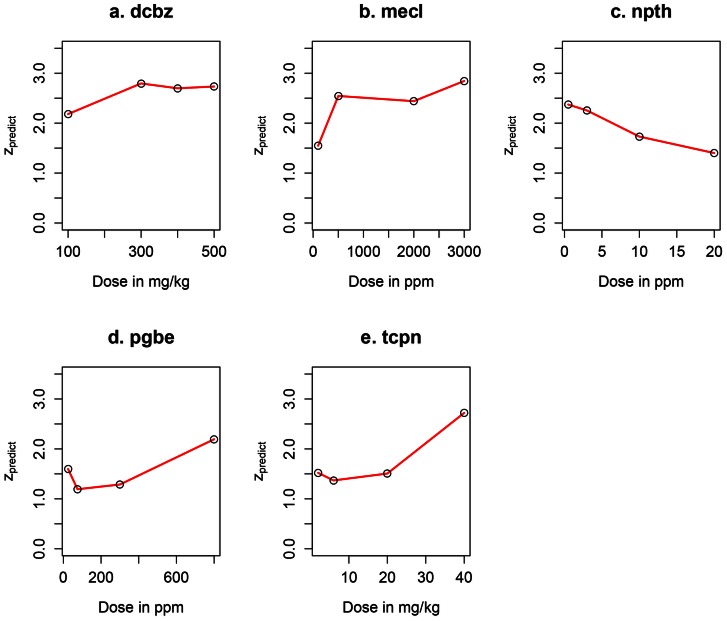
Dose-response predictions using the pathway-based prediction models for data of the chemicals tabulated in Table 2. Dose response predictions for five chemicals (a. dcbz, b. mecl, c. npth, d. pgbe and e. tcpn) treated at four different doses in mice.

The predicted dose response for the chemicals in Table 2 is shown in Figure 2. The slopes of the curves and the p-values of the alternate hypothesis that the slopes are positive are given in Table 6. NPTH is the only non-liver carcinogen among the five chemicals and had a negative slope. The responses derived from the model produce larger positive and more significant (lower p-values) slopes for the carcinogens. The fall in the carcinogenicity responses across the two lowest doses for PGBE and TCPN could be suggestive of alternate mode-of-actions at lower doses.

**Table 6:** Slopes of the dose-response curves in Figure 2.

Chemical	Slope^a^	p-value^b^
dcbz	0.137/100 mg/kg	0.09
mecl	0.158/500 ppm	0.12
npth	−0.256/5 ppm	0.99
pgbe	0.207/200 ppm	0.09
tcpn	0.331/10 mg/kg	0.05

a The slopes are in terms of changes in the predicted z values per change in tabulated dose. ^b^ p-value for hypothesis testing the alternative that the estimated slopes are positive.

### Conclusions

The analyses involved in this paper worked with hepatocarcinogenicity prediction models trained using data from mice and extrapolated to data sets from rats and humans. Alternate models derived from rats to predict carcinogenicity in other species could be attempted. This was not attempted here because of lack of sufficient analogous two-year cancer assay data for the rat and human data sets. The model derived here used data for chemicals at their maximum tolerable dose (MTD) levels. It could be argued that responses at lower doses are the ones with the most human relevance. Again this was not attempted here because of lack of sufficient number of chemicals dosed at lower levels. One possibility of understanding chemical-specific low-dose mechanisms would be the availability to a relatively large epidemiological data set of exposed people (e.g., [51] demonstrates an example of such a study for the case of workers exposed to benzene).

The mice used in the study lacked the genetic diversity of humans. So, the extrapolations of carcinogenicity predictions to human data has also to be viewed in the light of limitations in addressing concerns of identifying and characterizing the risk to susceptible populations. Populations could be considered susceptible either based on their genetics, age, nutritional and physical activity status and possibly other risk factors.

The B6C3F1 mice differ significantly from humans in the etiology of hepatocellular carcinoma (HCC). The mice have a relatively high background rate of developing HCC. In humans, HCC is thought to arise in a background of chronic inflammation, necrosis and regeneration, fibrosis and extracellular matrix deposition [52]. In comparison, HCC in mice are not known to arise under this background. Rather, a genetic event is assumed to precede a stepwise progression to HCC in mice. There are also known differences and similarities for the initiating genetic events for HCC between mice and humans. Therefore there is certainly a point in questioning the use of B6C3F1 mice when attempting to understand the etiology of human HCC. The utility of using the carcinogenicity results from existing rodent bioassays to the context of humans have been questioned by Bruce Ames and colleagues [53], [54], [55], [56]. This concern was in light of the relatively large proportion of tested chemicals being declared as rodent carcinogens. Some of these chemicals occur naturally in human dietary sources and others are being prescribed as pharmaceutical drugs. The exposures to humans from these sources were at doses much lower than what were used in the rodent bioassays. In fact the high positive rates from the rodent bioassays were hypothesized to have been caused by increased cell proliferation induced by the relatively high doses of the tested chemicals. Specifically for the data in this manuscript, none of the rodent liver carcinogens are currently known to be associated with human liver cancer.

However, in the predictive toxicology context of the manuscript, in spite of the known differences in etiologies, our hypothesis is that gene expression levels of the precancerous lesions are the similar in mice and humans. This hypothesis is validated by the relatively high predictability of human hepatocarcinogenicity using an independently derived predictive model using 90 day gene expression data from B6C3F1 mice. Further, the work in Hoeneroff et al suggests that this hypothesis is not without justification. In their work the authors found similar gene expression profiles in cells from HCCs obtained from mice and human samples. The situation is analogous to the case of azoxymethane induced colon cancer [57] in rodents being used as a model of human colon cancer. So the mode of action (chronic inflammation, increased proliferation or specific genetic events) leading to the pre-cancerous lesions in humans and rodents may be similar or different but once the precancerous state is reached then one could hypothesize that (at least evolutionarily) both species may follow similar paths to tumor formation and progression.

In summary, we rigorously derive and evaluate a biochemical pathway based hepatocarcinogenicity prediction model. Among the set of alternate prediction models , random forests were found to perform the best in terms of cross-validated risk. The model used gene expression data from a given tissue at the end of a short term study to predict the risk for development of tumors at the end of a longer period of time. Specifically, the model is evaluated using gene expression data obtained from the liver of mice treated with a range of 26 chemicals over a period of 90 days. The model with the information on affected pathways derived using these gene expression data had sufficient signal to adequately predict the two-year liver carcinogenicity risk of the same chemicals as evaluated in the National Toxicology Program's two-year cancer bioassay. The fact that the model was developed at the biochemical pathway level allows one to reasonably expect conserved behaviors of the chemicals at the pathway level across multiple species. This belief was validated using the model developed for mice to predict results in humans and rats. This fact was observed in Figure 1.The use of pathways in the carcinogenicity prediction model allowed a biologically based reduction of the feature space of the classifier. The responses of the various pathways suggested a complex interplay between them leading to the carcinogenicity prediction. What was encouraging was the excellent extrapolation for the case of human data.

## Supporting Information

Figure S1 **Clustergram of transformed p-values (** **Equation (1** **)) representing the enrichment of the 216 pathways across the 26 chemicals treatments in mice, was generated using hierarchical clustering with the euclidean distance metric and average linkage to generate the hierarchical trees of pathways and chemicals using the Cluster and Tree view programs** [**58**] **.**
(PNG)

Text S1 **Provides additional description of the microarray experiments, involved in mice and rats, the data for which are used in the analysis.** Also provided are details of generation of Figure S1, a discussion of the identified false positives and false negatives by the prediction models, primers on the technique of cross-validation , receiver-operator curves and references to the NTP technical reports of the two-year cancer bioassays for each of the 26 mice treated chemicals.
(DOC)

Table S1 **List of rat hepatocarcinogen treatment groups from Auerbach et al., 2009.**
(XLS)

Table S2 **List of chemicals in** **Table 1** **that were chosen to be in each of the 5 folds in the cross-validation analysis performed.**
(XLS)

Table S3 **List of various learning algorithms attempted in the SuperLearner package** [**35**] **and the cross-validated risk for the case of pathway-based continuous predictions.**
(XLS)

Table S4 **The list of pathways used as predictors along with their importance measures as reported by the random forests learning algorithm** [**37**] **.** The importance measures for each of the pathways are also reported.
(XLS)
