# Supplementary figures and images for: Biological Networks for Predicting Chemical Hepatocarcinogenicity Using Gene Expression Data from Treated Mice and Relevance across Human and Rat Species
Source: PLoS One. 2013 May 30;8(5):e63308. doi: 10.1371/journal.pone.0063308 (PMC3667849; doi:10.1371/journal.pone.0063308)

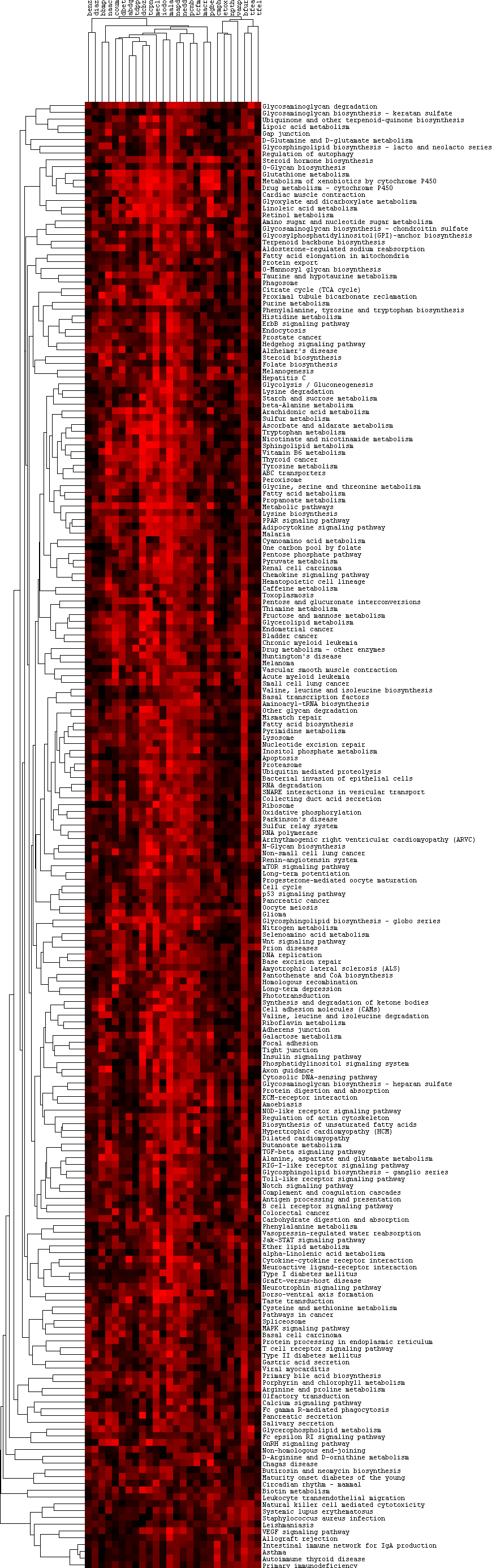

Supplement: Figure S1 — Clustergram of transformed p-values ( Equation (1 )) representing the enrichment of the 216 pathways across the 26 chemicals treatments in mice, was generated using hierarchical clustering with the euclidean distance metric and average linkage to generate the hierarchical trees of pathways and chemicals using the Cluster and Tree view programs [58] . (PNG) [file pone.0063308.s001.png]
