# Supplementary material for: Biological Networks for Predicting Chemical Hepatocarcinogenicity Using Gene Expression Data from Treated Mice and Relevance across Human and Rat Species
Source: PLoS One. 2013 May 30;8(5):e63308. doi: 10.1371/journal.pone.0063308 (PMC3667849; doi:10.1371/journal.pone.0063308)
Supplement: Text S1 — Provides additional description of the microarray experiments, involved in mice and rats, the data for which are used in the analysis. Also provided are details of generation of Figure S1, a discussion of the identified false positives and false negatives by the prediction models, primers on the technique of cross-validation , receiver-operator curves and references to the NTP technical reports of the two-year cancer bioassays for each of the 26 mice treated chemicals. (DOC) [file pone.0063308.s002.doc]

**Mouse liver gene expression experiments**

***Chemicals.*** 1,5-Naphthalenediamine (NAPD; CAS No. 2243-62-1; Purity: 97%), 2,3-benzofuran (BFUR; CAS No. 271-89-6; Purity: 99%), N-(1-naphthyl)ethylenediamine dihydrochloride (NEDD; CAS No. 1465-25-4; Purity: 98%), pentachloronitrobenzene (PCNB; CAS No. 82-68-8; Purity: 99%), 2,2-bis(bromomethyl)-1,3-propanediol (BBMP; CAS No. 3296-90-0; Purity: 98%), 1,2-dibromoethane (DBET; CAS No. 106-93-4; Purity: 99%), coumarin (COUM; CAS No. 91-64-5; Purity: 99%), benzene (BENZ; CAS No. 71-43-2; Purity: 99%), 2-chloromethylpyridine hydrochloride (CMPH; CAS No. 6959-47-3; Purity: 98%), 1,4-dichlorobenzene (DCBZ; CAS No. 106-46-7; Purity 99%), 1,2,3-trichloropropane (TCPN; CAS No. 96-18-4; Purity 99%), propylene glycol mono-*t*-butyl ether (PGBE; CAS No. 57018-52-7; Purity 99%), naphthalene (NPTH; CAS No. 91-20-3; Purity 99%), vanadium pentoxide (VANP; CAS No. 1314-62-1; Purity 99.6%), methylene chloride (MECL; CAS No. 75-09-2; Purity 99.5%), trichlorofluoromethane (TCFM; CAS No. 75-69-4; Purity: 99%), and iodoform (IODO; CAS No. 75-47-8; Purity: >99%) were purchased from Sigma-Aldrich (St. Louis, MO). 1-Amino-2,4-dibromoanthraquinone (ADBQ; CAS No. 81-49-2; Purity: 99%) was purchased from Apin Chemicals (Oxfordshire, United Kingdom). Tris(2,3-dibromopropyl)phosphate (TDPP; CAS No. 126-72-7; Purity: >95%) was purchased from Narchem Corporation (Chicago, IL). Tetrafluoroethylene (TFEL; CAS No. 116-14-3) was purchased from E.I. du Pont de Nemours and Company (Wilmington, DE). Tetrafluoroethane (TFEA; CAS No. 811-97-2; Purity: 99%) was provided by Honeywell Corporation (Morristown, NJ). N-methylolacrylamide (MACR; CAS No. 924-42-5; Purity: 98%) was purchased from Pfaltz & Bauer (Waterbury, CT). 4-Nitroanthranilic acid (NAAC; CAS No. 619-17-0; Purity: 98.5%), diazinon (DIAZ; CAS No. 333-41-5; Purity: 98%), and malathion (MALA; CAS No. 121-75-5; Purity: 95%) were purchased from Advanced Technology and Industry (Hong Kong, China). Ethylene oxide (ETOX; CAS No. 75-21-8) was purchased from Linde Gas (Cleveland, OH). The above list of chemicals and their associated descriptions are tabulated in Table 1.

***Animals and Treatment.*** Female B6C3F_1_ mice were obtained from Charles River Laboratories (Raleigh, NC). Twenty-five of the 26 chemicals used in this study have been previously tested by the NTP and the results are summarized in Table 1. The remaining chemical, tetrafluoroethane, was independently tested in a two-year rodent bioassay (Alexander et al. 1995). For each compound, 90-day animal studies were performed in multiple phases across multiple years using a matched vehicle control group run concurrently with the exposure. For all of these chemicals, at least one of the doses from the two-year bioassay was used for the 90-day studies. For five chemicals, methylene chloride, naphthalene, propylene glycol mono-t-butyl ether, 1,4-dichlorobenzene, and 1,2,3-trichloropropane, a five-point dose response with a matched control group (Table 2) was used. The concentrations for these exposures overlapped those in the original NTP rodent bioassays.

Mice were housed in a temperature (17.8 to 26.1°C) and humidity (30 to 70%) controlled environment with a standard 12 h light/dark cycle. Air flow within the housing environment was maintained at 12 to 15 air changes per hour. The animals were provided access to water purified by reverse osmosis and certified ground NIH-07 food (Zeigler Brothers, Inc., Gardners, PA) *ad libitum* except for animals exposed via inhalation where food and water were withheld during exposures. At the beginning of each study, the mice were randomized by weight and divided into treatment groups. Animal treatment was initiated at 5 to 6 weeks of age and were performed via the route and dose listed in Table 1. Animals were exposed for a total of 13 weeks. For food and gavage exposures, mice were housed 5 per cage in polycarbonate cages. Gavage exposures were administered 5 days per week and feed exposures were provided 7 days per week. For inhalation exposures, mice were exposed for 6 hr per day, 5 days per week in either 1 m^3^ whole body inhalation chambers (Hazelton H1000, Lab Products, Seaford, DE) or single animal whole body chambers (Wong et al. 2008) For animals exposed in the 1 m^3^ whole body inhalation chambers, animals were also housed individually in the chamber during non-exposure periods. For animal exposed in the single animal whole body chambers, animals were placed into the chambers prior to the start of exposure and then removed from the chambers and returned to domiciliary housing (5 animals per cage) after the end of the 6-hour exposure.

Animal use in this study was approved by the Institutional Animal Use and Care Committee of The Hamner Institutes for Health Sciences and was conducted in accordance with the National Institutes of Health guidelines for the care and use of laboratory animals. Animals were housed in fully-accredited American Association for Accreditation of Laboratory Animal Care (AAALAC) facilities.

***Necropsy.*** Following 13 weeks of exposure, the mice were euthanized with a lethal *i.p.* dose of sodium pentobarbital (Abbott Laboratories, Chicago, IL). The right, caudate and median liver lobes were removed and minced together in RNA*later*™ (Ambion, Austin, TX). The histological changes observed are described in the supplementary material.

***Histological Changes.*** Gross histological examination of the liver tissue identified treatment-related lesions in BFUR, DCBZ, PGBE, MECL, NPTH, and MALA exposed mice. In the BFUR treatment group, increased cytoplasmic vacuolation was observed in all the mice examined. In the DCBZ treatment group, increased centrilobular hypertrophy was observed in mice treated with 400 mg/kg and above. The number of affected animals was eight out of eight mice examined in the 400 mg/kg group, eight out of nine mice examined in the 500 mg/kg group, and nine out of nine animals examined in the 600 mg/kg group. In the PGBE treated mice, centrilobular hypertrophy was noted in seven out of ten animals examined in the 1,200 ppm exposure group. No animals showed the changes at lower concentrations. In MECL treated mice, vacuolation was observed in the livers of mice exposed to 2,000 ppm and above. The vacuolation was characterized by variably sized vacuoles within the cytoplasm of hepatocytes and it was more pronounced in periportal areas. In the periportal areas, hepatocytes were distended with large clear vacuoles which frequently displaced the nuclei. The number of affected animals was nine out of ten mice examined in the 2,000 ppm group, ten out of ten mice examined in the 3,000 ppm group, and eight out of ten mice examined in the 4,000 ppm group. In NPTH exposed mice, centrilobular hypertrophy was observed in eight out of ten animals examined at the 30 ppm concentration. No hypertrophy was observed at lower concentrations. In MALA treated mice, hepatocellular hypertrophy was observed in the periportal region in all ten animals examined.

**Mice gene gxpression microarray analysis.** Microarray analysis was performed on 3 to 5 animals per treatment or concurrent vehicle control group. A total of 242 animals were analyzed for liver gene expression. Total RNA was isolated using Trizol reagent (Invitrogen, Carlsbad, CA). The isolated RNA was further purified using RNeasy columns (Qiagen, Valencia, CA) and the integrity of the RNA was verified spectrophotometrically and with the Agilent 2100 Bioanalyzer (Palo Alto, CA) (Suppl. Table 2). Double-stranded cDNA was synthesized from 5 μg of total RNA using the One-Cycle cDNA synthesis kit (Affymetrix, Santa Clara, CA). Biotin-labeled cRNA was transcribed from the cDNA using the GeneChip IVT Labeling Kit (Affymetrix). Fifteen μg of labeled cRNA was fragmented and hybridized to Affymetrix Mouse Genome 430 2.0 arrays for 16 hours at 45°C. The hybridized arrays were washed using the GeneChip Fluidics Station 450 and scanned using a GeneChip 3000 scanner. The gene expression results have been deposited in the National Center for Biotechnology Information Gene Expression Omnibus (Accession No.: GSE18858). The Affmetrix .CEL files for each pair of the twenty treatments and their matching vehicle controls were normalized separately using the GCRMA ((Wu et al. 2004)) function in R ((Team 2009)).

**Rat liver gene expression experiments**

The gene expression studies included vehicle controls and 90-day exposures to 1-Amino-2,4-dibromoanthraquinone, Aflatoxin B1, Acetominophen, Tryptophan and Methyeugenol. Tumor data came from the associated NTP bioassays. The animals were treated with these chemicals at the respective doses given in Table 3 through 90 days of their lives and were sacrificed on the 91^st^ day in order to extract liver tissue. There were six animals per treatment group.

Approximately 130 to 150 mg of liver tissue was used for each RNA extraction. After extraction the RNAs were resuspended in RNAse –free water and concentrations were measured using a spectrophotemeter. Integrity of RNA samples from both studies was determined by the size distribution of the 18s and 28S ribosomal RNA using an Agilent Bioanalyzer (Agilent Technologies, Palo Alto, CA). In both studies all samples passed quality control and showed no signs of degradation. For microarray hybridization, five hundred nanograms of total RNA was converted into labeled cRNA with nucleotides coupled to fluorescent dye Cy3 using the Low RNA Input Linear Amplification Kit (Agilent Technologies, Palo Alto, CA) following the manufacturer’s protocol. The A260/280 ratio and yield of each of the cRNAs was determined and cRNA integrity was evaluated using an Agilent Bioanalyzer. nCy3-labeled cRNA (1.65 ng) from each sample was hybridized to Agilent Rat Whole Genome Oligonucleotide Microarrays in 4X44K format. The hybridized array was then washed and scanned and data was extracted from the scanned image using Feature Extraction version 9.5 (Agilent Technologies). All microarrays passed QC standards set forth in the Agilent feature extraction software (V9.5) reference guide. The gProcessed signal from the Agilent 4X44K arrays used in both the CVNC and FA study data was normalized using quantile normalization followed by per chip median centering. The data from CVNC and FA study was normalized separately.

**Missing gene data**

The analyses described include gene expression data from multiple microarray platforms. Not all microarray platforms considered in this study have probes for all the KEGG pathway genes. On average a microarray platform had data for 80% of the KEGG pathway genes. In situations where the platform misses a KEGG pathway gene, then the gene is assumed to not change under the experimental conditions. This assumption is a limitation but is reasonable given the relatively large coverage of the KEGG pathway genes.

Table: The datasets and number of KEGG pathway genes covered by the probes on the various microarray platforms used in this study.

| **GEO id** | **Organism** | **No of KEGG pathway genes** | **No of KEGG path genes on microarray** | **Platform** |
| --- | --- | --- | --- | --- |
| GDS2239 | Human | 5700 | 4741 | [HG-U133A] Affymetrix Human Genome U133A Array |
| GDS3347 | Human | 5700 | 3964 | [HG_U95Av2] Affymetrix Human Genome U95 Version 2 Array |
| GDS3656 | Human | 5700 | 5316 | Sentrix HumanRef-8 Expression BeadChip |
| GSE10356 | Human | 5700 | 1931 | INSERM Homo sapiens 14K array Liverpool3 |
| GSE15331 | Human | 5700 | 5332 | Agilent-012097 Human 1A Microarray (V2) G4110B |
| GSE15653 | Human | 5700 | 4735 | [HG-U133A] Affymetrix Human Genome U133A Array |
| GSE16415 | Human | 5700 | 5192 | ABI Human Genome Survey Microarray Version 2 |
| GSE20948 | Human | 5700 | 5320 | [HG-U133_Plus_2] Affymetrix Human Genome U133 Plus 2.0 Array |
| GSE23343 | Human | 5700 | 5320 | [HG-U133_Plus_2] Affymetrix Human Genome U133 Plus 2.0 Array |
| Mice data | Mouse | 6461 | 5197 | Affymetrix Mouse Genome 430 2.0 arrays |
| Rat data | Rat | 6413 | 5338 | Agilent Rat Whole Genome Oligonucleotide Microarrays in 4X44K format |

**Generation of clustergram**

Euclidean distance represents a definition of the degree of closeness between the different pathways or different chemicals based on their responses across the various pathways. Roughly, average linkage represents the criteria of assigning a new member (chemical or pathway) to a cluster if the average distance between the new member and the existing members of the cluster is small. The clustergram of transformed p-values (Equation (1)) representing the enrichment of the 216 pathways across the 26 chemicals treatments in mice, was generated using hierarchical clustering with the euclidean distance metric and average linkage to generate the hierarchical trees of pathways and chemicals using the Cluster and Tree view programs (Eisen et al. 1998). The clusters derived are hierarchical in the sense that the various clusters can be organized hierarchically i.e, the individual chemicals or pathways can be paired, pairs combined with individual members to forms clusters of triplets or pairs of chemicals can combined with other pairs and so on to form new clusters.

**Discussion of false positives and false negatives as identified by the prediction models for mouse, rat and human liver carcinogenicity**

For PCNB, the carcinogenicity study was conducted by the National Cancer Institute (NCI) prior to the formation of the NTP and did not follow the current design. This study lasted only 92 weeks with an increasing dose from 4640 ppm for first four weeks and 9000 ppm from week 32 to week 77 with the last 15 weeks receiving no PCNB. Thus, our prediction may be correct had the current NTP design of two years constant exposure been used. DBET was one of the earlier bioassays done by the NCI with marked survival reduction (study was terminated at 75 weeks), probably from a very strong finding of metastatic squamous cell carcinomas arising from the forestomach. The reduced survival seriously limits the value of this study for liver cancer and this probably should redone at a lower exposure to confirm or refute our prediction. For COUM, female mice were positive for liver adenomas based on NTPs analysis, but not for adenomas and carcinomas combined. This analysis suggests that COUM should be considered a positive for liver tumors since the same mechanisms appear to be active as for the other liver tumors. Similar problems were present for the NEDD carcinogenicity study. For the remaining four (MALA, IODO, TCFM and NAAC) nothing in the reports indicates pre-neoplastic or neoplastic effects in the liver so these appear to be false positive. The false positives (APAP, VtC and TYP) in the case of rats may represent the limitation of the extrapolation from mice.

With the exception of TFEL, all of the other chemicals produced liver tumors consisting primarily of hepatocellular adenomas and carcinomas. For TFEL, hemangiomas and hemangiosarcomas were the primary tumor types in the liver suggesting it may work through a different mechanism. The false negatives in the case of rats were all associated with three carcinogenic chemicals at their lower doses suggesting either a noisy informative signal for these chemical-dose combinations or different mechanisms at lower doses.

For the case of human data, all false positives were related to gene polymorphisms associated with cancer data sets. The false negative was for the Type 2 diabetes data set (GSE16415) where the gene expression was derived from the adipose tissue. This suggests possible alternate mechanisms in the adipose tissue versus the liver from which the model was inferred.

**References to the NTP technical reports for the mice treatments**

The references to the technical reports for the NTP’s two year bioassay for the chemical treatments are given in (1978a, b, c, d, e, f, g, 1979a, b, c, 1983, 1986a, b, 1987a, b, c, 1989a, b, 1992, 1993a, b, 1996a, b, 1997, 2000, 2002, 2004)

**Primers on cross-validation and receiver-operator curves**

**Cross-validation**: This is a technique typically employed in the context of statistical prediction/learning models where the goal is to predict an outcome for a given input. In particular for this paper, hepatocarcinogenicity is the outcome of interest and the given input is the biochemical pathway responses of a given species when treated with a given dose of a particular chemical. In general, the number of features of the input is much larger than the number of data points available to fit the model. For this paper, there were 216 pathway responses associated with each of 26 chemicals. So it is very easy to over fit a given prediction model. One would really like to get the right prediction for a new input to the model. Cross-validation is a technique that is used to estimate the expected prediction error for such new inputs to the prediction model. It works by dividing the given input set into *k* sets, known as *k*-fold (5-fold for this paper) cross-validation. The method then tests the predictions on each of these *k* sets, using a model that was trained on data from the remaining *k*-1 sets. This allows for a choice of a model that is not over-fit and also provides an estimate of the expected prediction error.

**Receiver-operator curves**: These are essentially performance evaluation tools for a prediction algorithm/model. A lot of you may be more aware of the issue of multiple testing in the context of statistical hypothesis tests. Your choice of the p-value cut-off is a trade-off between the number of discoveries you make and proportion of false ones among those. For ROC curve generation, you pick a subset of positives and negatives from a space in which you want your prediction method to work. The difference with the multiple testing scenarios is that in this situation you assume that you know what the positives and the negatives are. If you are really lazy then your predictor could be a coin toss. This is how a coin toss predictor would behave on an ROC curve. An important measure of the ROC curve is the area under the curve (AUC). For the coin toss the AUC is 0.5. Predictor 1 is better than the coin toss, predictor 2 is better than predictor 1 and finally we have the perfect predictor. These three predictors are shown in an ROC curve below.


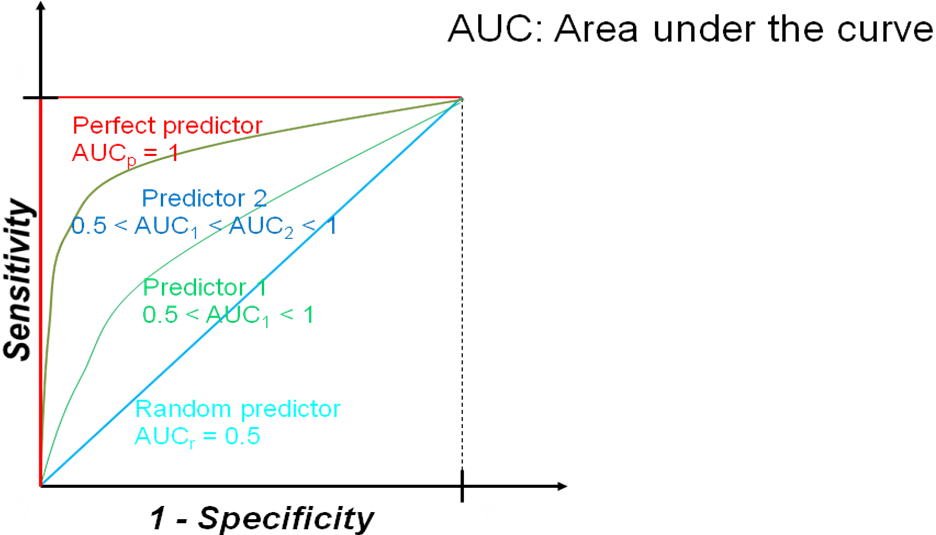


**References**

Alexander DJ, Libretto SE, Chevalier HJ, Imamura T, Pappritz G, Wilson J. 1995. HFA-134a (1,1,1,2-tetrafluoroethane); lack of oncogenicity in rodents after inhalation. Hum Exp Toxicol 14(9): 706-714.

**Wong BA, Ross PW, James RA. 2008. Development and use of a single-animal whole-body system for inhalation exposure. Lab animal 37(1): 33-40.**

**Wu Z, Irizarry RA, Gentleman R, Martinez-Murillo F, Spencer F. 2004. A model-based background adjustment for oligonucleotide expression arrays. Journal of the American Statistical Association 99(468): 909-917.**

**Team RDC. 2009. R: A Language and Environment for Statistical Computing. R Foundation for Statistical Computing.**

**Eisen M, Spellman P, Brown P, Botstein D. 1998. Cluster analysis and display of genome-wide expression patterns. Proceedings of the National Academy of Sciences of the United States of America 95(25): 14863.**

**[Anonymous]. 1978a. Bioassay of 1,5-naphthalenediamine for possible carcinogenicity. Natl Cancer Inst Carcinog Tech Rep Ser 143: 1-123.**

**[Anonymous]. 1978b. Bioassay of tris (2,3-dibromopropyl) phosphate for possible carcinogenicity. Natl Cancer Inst Carcinog Tech Rep Ser 76: 1-123.**

**[Anonymous]. 1978c. Bioassay of 1,2-dibromoethane for possible carcinogenicity. Natl Cancer Inst Carcinog Tech Rep Ser 86: 1-129.**

**[Anonymous]. 1978d. Bioassay of iodoform for possible carcinogenicity (CAS No. 75-47-8). Natl Cancer Inst Carcinog Tech Rep Ser 110: 1-107.**

**[Anonymous]. 1978e. Bioassay of 4-nitroanthranilic acid for possible carcinogenicity (CAS No. 619-17-0). Natl Cancer Inst Carcinog Tech Rep Ser 109: 1-123.**

**[Anonymous]. 1978f. Bioassay of trichlorofluoromethane for possible carcinogenicity (CAS No. 75-69-4). Natl Cancer Inst Carcinog Tech Rep Ser 106: 1-99.**

**[Anonymous]. 1978g. Bioassay of L-Tryptophan for Possible Carcinogenicity (CAS No. 73-22-3). Natl Toxicol Program Tech Rep Ser 71: 1-115.**

**[Anonymous]. 1979a. Bioassay of diazinon for possible carcinogenicity. Natl Cancer Inst Carcinog Tech Rep Ser 137: 1-115.**

**[Anonymous]. 1979b. Bioassay of Malathion for Possible Carcinogenicity (CAS No. 121-75-5). Natl Toxicol Program Tech Rep Ser 192: 1-87.**

**[Anonymous]. 1979c. Bioassay of N-(1-naphthyl)ethylenediamine dihydrochloride for possible carcinogenicity. Natl Cancer Inst Carcinog Tech Rep Ser 168: 1-103.**

**[Anonymous]. 1983. NTP Carcinogenesis Bioassay of L-Ascorbic Acid (Vitamin C) (CAS No. 50-81-7) in F344/N Rats and B6C3F1 Mice (Feed Study). Natl Toxicol Program Tech Rep Ser 247: 1-172.**

**[Anonymous]. 1986a. NTP Toxicology and Carcinogenesis Studies of Dichloromethane (Methylene Chloride) (CAS No. 75-09-2) in F344/N Rats and B6C3F1 Mice (Inhalation Studies). Natl Toxicol Program Tech Rep Ser 306: 1-208.**

**[Anonymous]. 1986b. NTP Toxicology and Carcinogenesis Studies of Benzene (CAS No. 71-43-2) in F344/N Rats and B6C3F1 Mice (Gavage Studies). Natl Toxicol Program Tech Rep Ser 289: 1-277.**

**[Anonymous]. 1987a. NTP Toxicology and Carcinogenesis Studies of Ethylene Oxide (CAS No. 75-21-8) in B6C3F1 Mice (Inhalation Studies). Natl Toxicol Program Tech Rep Ser 326: 1-114.**

**[Anonymous]. 1987b. NTP Toxicology and Carcinogenesis Studies of 1,4-Dichlorobenzene (CAS No. 106-46-7) in F344/N Rats and B6C3F1 Mice (Gavage Studies). Natl Toxicol Program Tech Rep Ser 319: 1-198.**

**[Anonymous]. 1987c. NTP Toxicology and Carcinogenesis Studies of Pentachloronitrobenzene (CAS No. 82-68-8) in B6C3F1 Mice (Feed Studies). Natl Toxicol Program Tech Rep Ser 325: 1-122.**

**[Anonymous]. 1989a. NTP Toxicology and Carcinogenesis Studies of Benzofuran (CAS No. 271-89-6) in F344/N Rats and B6C3F1 Mice (Gavage Studies). Natl Toxicol Program Tech Rep Ser 370: 1-189.**

**[Anonymous]. 1989b. NTP Toxicology and Carcinogenesis Studies of N-Methylolacrylamide (CAS No. 924-42-5) in F344/N Rats and B6C3F1 Mice (Gavage Studies). Natl Toxicol Program Tech Rep Ser 352: 1-204.**

**[Anonymous]. 1992. Toxicology and Carcinogenesis Studies of Naphthalene (CAS No. 91-20-3) in B6C3F1 Mice (Inhalation Studies). Natl Toxicol Program Tech Rep Ser 410: 1-172.**

**[Anonymous]. 1993a. NTP Toxicology and Carcinogenesis Studies of Coumarin (CAS No. 91-64-5) in F344/N Rats and B6C3F1 Mice (Gavage Studies). Natl Toxicol Program Tech Rep Ser 422: 1-340.**

**[Anonymous]. 1993b. NTP Toxicology and Carcinogenesis of 1,2,3-Trichloropropane (CAS No. 96-18-4) in F344/N Rats and B6C3F1 Mice (Gavage Studies). Natl Toxicol Program Tech Rep Ser 384: 1-348.**

**[Anonymous]. 1996a. NTP Toxicology and Carcinogenesis Studies of 1-Amino-2,4-Dibromoanthraquinone (CAS No. 81-49-2) in F344/N Rats and B6C3F1 Mice (Feed Studies). Natl Toxicol Program Tech Rep Ser 383: 1-370.**

**[Anonymous]. 1996b. NTP Toxicology and Carcinogenesis Studies of 2,2-Bis(Bromomethyl)-1,3-Propanediol (FR-1138(R)) (CAS No. 3296-90-0) in F344 Rats and B6C3F1 Mice (Feed Studies). Natl Toxicol Program Tech Rep Ser 452: 1-465.**

**[Anonymous]. 1997. NTP Toxicology and Carcinogenesis Studies of Tetrafluoroethylene (CAS No. 116-14-3) in F344 Rats and B6C3F1 Mice (Inhalation Studies). Natl Toxicol Program Tech Rep Ser 450: 1-321.**

**[Anonymous]. 2000. NTP Toxicology and Carcinogenesis Studies of Methyleugenol (CAS NO. 93-15-2) in F344/N Rats and B6C3F1 Mice (Gavage Studies). Natl Toxicol Program Tech Rep Ser 491: 1-412.**

**[Anonymous]. 2002. NTP toxicology and carcinogensis studies of vanadium pentoxide (CAS No. 1314-62-1) in F344/N rats and B6C3F1 mice (inhalation). Natl Toxicol Program Tech Rep Ser(507): 1-343.**

**[Anonymous]. 2004. NTP technical report on the toxicology and carcinogenesis studies of propylene glycol mono-t-butyl ether (CAS No. 57018-52-7) in F344/N rats and B6C3F1 mice and a toxicology study of propylene glycol mono-t-butyl ether in male NBR rats (inhalation studies). Natl Toxicol Program Tech Rep Ser(515): 1-306.**
